# Electronic Biosensing with Functionalized rGO FETs

**DOI:** 10.3390/bios6020017

**Published:** 2016-04-22

**Authors:** Ciril Reiner-Rozman, Caroline Kotlowski, Wolfgang Knoll

**Affiliations:** 1 Center for Electrochemical Surface Technology (CEST), Wiener Neustadt 2700, Austria; rozman.ciril.fl@ait.ac.at (C.R.-R.); caroline.kotlowski@boku.ac.at (C.K.); 2 AIT Austrian Institute of Technology, Wien 1220, Austria

**Keywords:** reduced graphene oxide (rGO graphene), FET, liquid-gate, biosensing, receptor immobilization, antigen-antibody interaction, food toxins, aflatoxins, odorant-binding proteins, olfaction, smell sensor

## Abstract

In the following we give a short summary of examples for biosensor concepts in areas in which reduced graphene oxide-based electronic devices can be developed into new classes of biosensors, which are highly sensitive, label-free, disposable and cheap, with electronic signals that are easy to analyze and interpret, suitable for multiplexed operation and for remote control, compatible with NFC technology, *etc.*, and in many cases a clear and promising alternative to optical sensors. The presented areas concern sensing challenges in medical diagnostics with an example for detecting general antibody-antigen interactions, for the monitoring of toxins and pathogens in food and feed stuff, exemplified by the detection of aflatoxins, and the area of smell sensors, which are certainly the most exciting development as there are very few existing examples in which the typically small and hydrophobic odorant molecules can be detected by other means. The example given here concerns the recording of a honey flavor (and a cancer marker for neuroblastoma), homovanillic acid, by the odorant binding protein OBP 14 from the honey bee, immobilized on the reduced graphene oxide gate of an FET sensor.

## 1. Introduction

The detection and quantitative monitoring of biomolecules in air, in water, in complex liquids like bodily fluids or similar is still a challenge, both from a fundamental point of view as well as in the context of practical applications. Whether it is the need for sensors in general air management scenarios—e.g., to detect air pollutants (including explosives), to sense crop disease markers, in food quality management for the identification of toxins, or in medical applications like breath analysis—Or for the detection of certain markers in blood, plasma, saliva, urine, wound liquids, *etc.*, that report a patient’s health and/or disease status, in all cases we are dealing with three major problems; (i) the sensitivity of the technical device for the quantification of a particular molecule of interest; (ii) the selectivity needed to differentiate between similar molecules; and (iii) the suppression of non-specific interactions with and binding to the sensor surface by other components in the sample volume, typically in excess to the actually targeted molecule.

Numerous concepts and formats for sensors based on optical transduction principles have been reported in the literature, some of which even made it to the market place and are commercialized [1]. Not so widespread are sensors based on electrical, electrochemical or electronic transduction principles [2]. The latter category, in particular, is the youngest member of the huge family of current-based bio-sensors; typical examples are CMOS-transistor-based devices that have been used to monitor action potentials of excited neurons [3] or cardiomyocytes [4] grown on the gate surface of the device, Si-nanowire based transistor read-out of protein and DNA binding [5], or the promising use of organic (“plastic”) electronics for the development of cheap and, hence, disposable sensors [6,7]. The most recent development in this area, the use of the novel carbon materials, *i.e.*, carbon nanotubes or graphene, opens a totally new but very promising range of options for biosensors based on electronic devices made from these highly interesting materials [8].

In this short summary, we will report some of our own efforts in designing, fabricating, bio-functionalizing and characterizing biosensors based on reduced graphene oxide as the gate material. We will give a very brief overview of the basic preparation protocols, and describe the electronic performance of the devices as transistors [9]. The practical examples given are (i) the detection of a standard biomolecule, bovine serum albumin (BSA), binding from solution to its antibody, immobilized on the sensor surface; (ii) the detection and quantification of food toxins (mycotoxins) binding to their antibodies; and (iii) the monitoring of an odorant molecule (dissolved in an aqueous solution) to the surface-bound odorant binding protein from an insect, the honey bee *Apis mellifera* [10].

## 2. Preparation and General Electronic Performance of rGO-FET Biosensors

**Figure 1:**
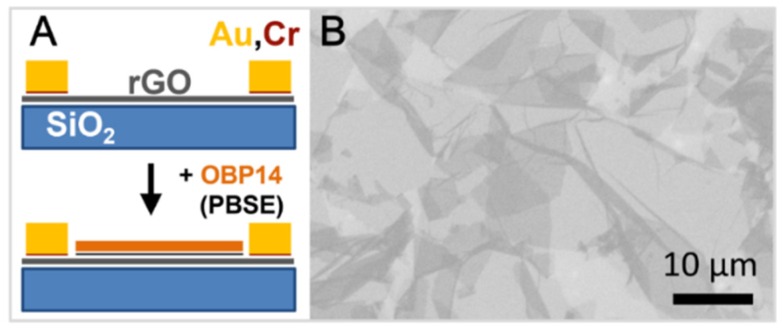
(**A**) Schematic illustration of the individual fabrication steps of the graphene biosensor device: After the reduction of the GO flakes to rGO, source and drain Au electrodes were evaporated (with a thin layer of Cr as an adhesion promotor), then coated (via self-assembly) by a linker, PBSE, and finally functionalized by the attachment of antibodies or by odorant binding proteins, here OBP 14 from the honey bee; (**B**) Scanning electron microscopic image of the GO flakes on the chip substrate (before reduction to rGO and before being coated with the electrodes).

Reduced graphene oxide field-effect transistor (rGO-FET) devices were fabricated with a typical channel width of 40 µm. Figure 1A briefly summarizes the individual preparation steps for the devices [10]. Silicon wafers with a 300 nm oxide layer were chosen as base substrates. They were cleaned following a standard RCA cleaning procedure and then submerged in a 1%–2% 3-amino-propyl-triethoxy-silane-(APTES) solution in ethanol for 1 h, with APTES forming a monolayer on the substrate that increases the adhesion of graphene (reduced graphene oxide).

Graphene oxide sheets were prepared using a variation of Hummers method [11,12]. The obtained graphene oxide flakes were deposited onto Si-wafers via spin coating. A scanning electron microscopic image of the GO flakes on the substrate before reduction of rGO and before the evaporation of the Au electrodes for the transistor are shown in Figure 1B. After rinsing with ethanol, the substrates were heated to 120 °C for two hours and then cooled to room temperature. They were then treated overnight in hermetically sealed glass petri dishes with hydrazine at 70 °C in order to reduce the graphene oxide, forming the graphene structure consisting of sp^2^-hybrid bonds.

For the attachment of the antibodies (here, antibodies against bovine serum albumin, BSA, and aflatoxin B1, respectively) to the sensing area, the graphene surface was chemically modified by a bi-functional linker, 1-pyrenebutanoic acid succinimidyl ester (PBSE). One end the linker firmly attaches to the graphene surface through π-π interactions with the pyrene group while the other hand covalently reacts with one of the amino group of the protein to form an amide bond. All reagents were purchased from Sigma-Aldrich unless otherwise indicated and used without further purifications.

For the detection of different odorants, the biosensors were functionalized with an odorant binding protein 14 (OBP 14) from the honey Bee, *Apis mellifera*. The protein was expressed in bacterial systems using established protocols [13,14]. Purification of the proteins was accomplished using a combination of conventional chromatographic techniques followed by a final gel filtration step on Superose-12 (GE-Healthcare) as previously described in standard protocols [14].

**Figure 2:**
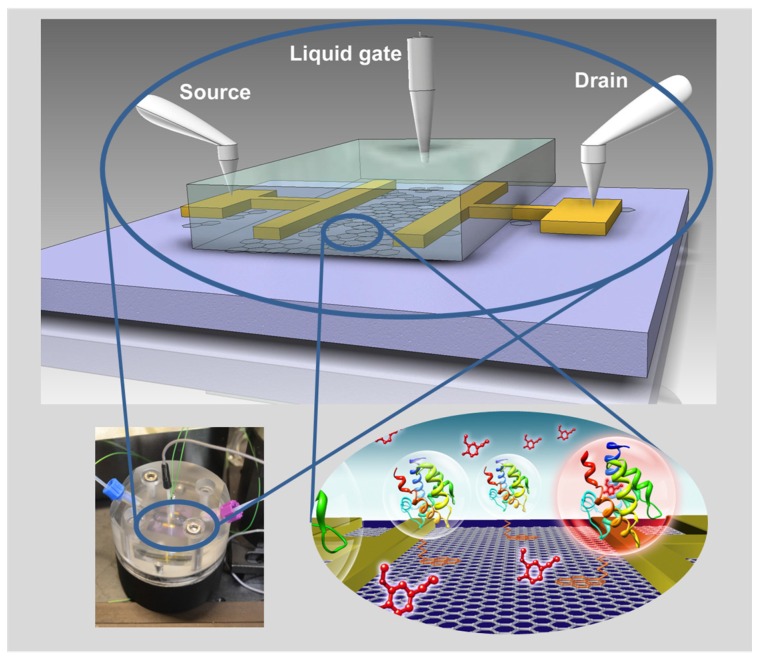
Schematics of the set-up and the device configuration consisting of a liquid gate electrode, source and drain electrodes, evaporated onto the reduced graphene oxide flakes on a solid Si/SiO_2_ substrate, functionalized by OBPs that are covalently coupled via a linker molecule. In the lower left corner is a photograph of the mounted flow cell.

The chip was then mounted into a flow cell for *in situ* real-time kinetic measurements (in order to quantify the association, k**_on_**, and dissociation rate constants, k**_off_**, respectively) as well as for titration experiments for the determination of affinity, K**_A_**, and dissociation constants, K**_d_**, respectively. An artist’s sketch of the FET setup and the immobilized OBP interacting with the odorant ligand, together with a photograph of the whole flow cell, are given in Figure 2.

**Figure 3:**
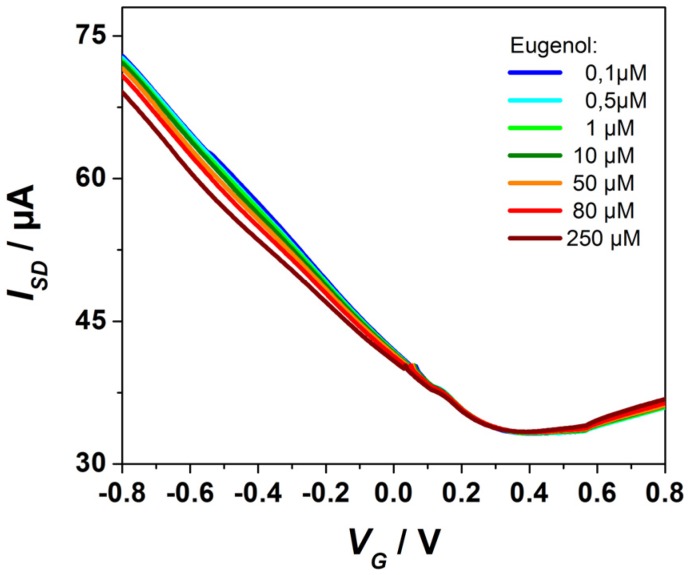
Source-drain current I_SD_-vs-gate voltage V_G_ characteristics of the OBP14 functionalized rGO FET-biosensor device at different Eugenol concentrations (as indicated) flowing through the cell.

The electrical properties of the FET devices were tested as described before [15]. Electrical measurements were performed using a Keithley 4200 semiconductor characterization system. An Ag/AgCl reference electrode (Flexref, World Precision Instruments) was used to operate the FET device in a liquid gate configuration with a constant source-drain bias of V**_SD_** = 50 mV. The cathodic branches of the I**_SD_**-vs-V**_G_** scans (*cf.* Figure 3) are dominated by the hole mobility [9]. The Dirac voltage is seen at ca. V**_G_** = 0.4 V. Recording these I**_SD_**-vs-V**_G_** scans at different bulk solution conditions, in particular in aqueous solutions of different ligand concentrations, results in a distinct shift of the cathodic branch which we attribute to a slight modification of the dipolar layer with the OBP protein monolayer immobilized on the graphene gate surface upon partial binding of the ligands to the free binding sites in the OBP acting as receptors.

## 3. Antigen–Antibody Interaction and the Limit of Detection

**Figure 4:**
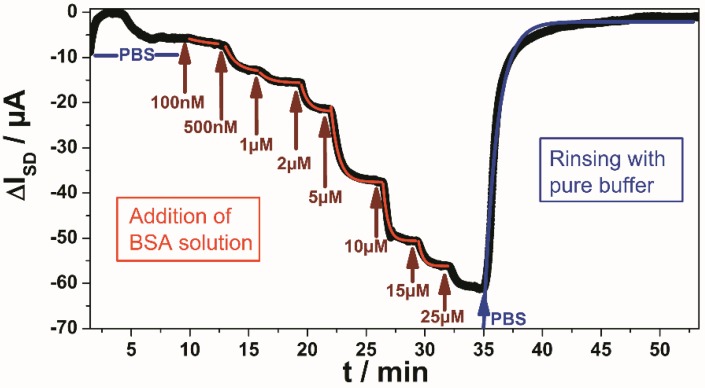
Global analysis of BSA binding from solution to surface-immobilized anti-BSA antibody; solutions with concentrations from 100 nM to (near) saturation at 25 μM (as indicated in red) were rinsed through the flow cell. The first few minutes show a drift due to the graphene charging behavior, which stabilizes after 7 min.

The first example that we describe for the use of these rGO-FETs as biosensors concerns the “classical” system, *i.e.*, the binding of the protein bovine serum albumin, BSA, as antigen to its FET-immobilized antibody. The global analysis protocol, *i.e.*, the stepwise increase of the bulk analyte concentration while simultaneously recoding the time-dependent change of the source-drain current, ΔI**_SD_**, is given in Figure 4. One can see that the current decreased each time the bulk concentration was increased from an initial concentration c_0_ = 100 nM, until it gradually reached a saturation level for a bulk concentration near c_0_ = 25 µM. The current decrease following each concentration change contains kinetic information (*cf.* the red curves that fit the current traces) occurs by a single exponential giving a time constant, k, that is concentration dependent (increases with bulk concentration). This is in agreement with the Langmuir model for this 1:1 complex between the analyte (the antigen) from solution and the surface–immobilized receptor (the antibody). Upon rinsing with pure buffer, the current returns to its original baseline level with a single exponential (*cf.* the blue fit curve) indicating the full reversibility of the binding event (a prerequisite for any analysis according the Langmuir model). The fit to this dissociation process results in a quantitative measure of the dissociation rate constant, k**_off_**. From the slope of the plot of all the rate constants determined as a function of the bulk concentration, one obtains the association rate constant, k**_on_**, which together with k**_off_** gives the affinity constant, K**_A_**. This has been documented recently and reported to result in an affinity constant for the binding of BSA to this antibody of K**_A_** = 1.6 × 10^5^ M^−1^ [9], corresponding to a dissociation constant of K**_d_** = 6.2 µM.

**Figure 5:**
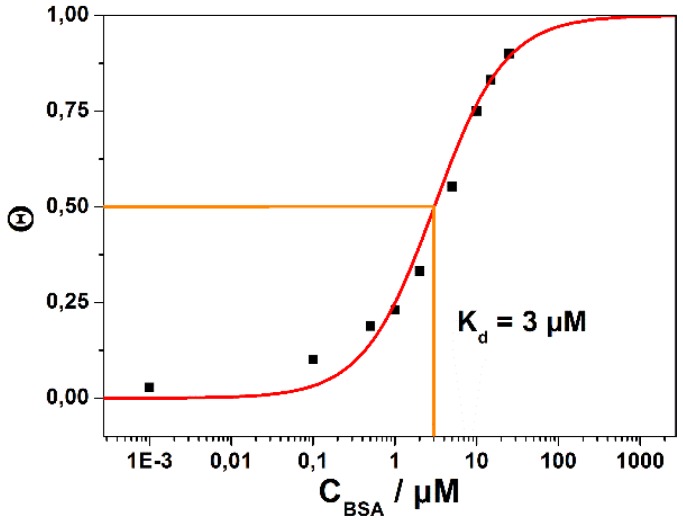
Langmuir adsorption isotherm obtained by plotting the (negative) change in the source-drain current, ΔI**_SD_**, as obtained from Figure 4 from the new stationary current after changing the concentration in the bulk solution to a new value. The red curve is a fit to the data according to the Langmuir model, resulting in a dissociation constant (half saturation concentration) K**_d_** = 3 µM.

Figure 5 summarizes the results taken from Figure 4, focusing here on the titration of the equilibrium surface coverage (*i.e*., the new source-drain current that one reads after the new equilibrium has been established) as a function of the bulk analyte concentration in solution, *i.e.*, the Langmuir isotherm. By plotting the surface coverage, Θ, *i.e.*, the source-drain current at a given concentration, I**_SD_** (c**_0_**), divided by I**_SD_** (c**_∞_**), the source drain current at infinite bulk analyte concentration, one obtains the affinity constant, K**_A_**:

Θ = I**_SD_** (c**_0_**)/I**_SD_** (c**_∞_**) = K**_A_** c**_0_**/(1 + K**_A_** c**_0_**) (1)

In Figure 5, we have plotted the surface coverage as a function of the (logarithm of the) bulk analyte concentration, and obtain the expected S-shaped curve. The corresponding fit to the data (full red curve in Figure 5) results in an affinity constant K**_A_** = 3.3 × 10^5^ M^−1^, corresponding to a dissociation constant, *i.e.*, a half-saturation constant, K**_d_** = 3 µM. Compared to the value obtained from the kinetic measurements (K**_d_** = 6.2 µM) this can be considered as an excellent confirmation of the applicability of the Langmuir model to the quantitative description of the binding assay of BSA to its surface-immobilized antibody. Furthermore, it confirms that the electronic read-out of this model binding reaction can be considered as a quantitative method for general biosensing purposes.

**Figure 6:**
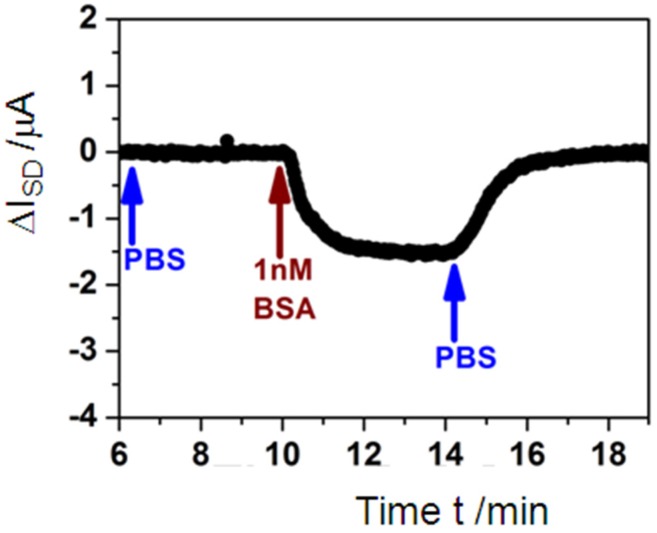
Single kinetic run, *i.e.*, recording of the change in the source-drain current, ΔI**_SD_**, after switching in the flow cell from pure PBS buffer (blue arrow) to a 1 nM BSA solution (brown arrow) in order to monitor the association rate constant, and back to pure buffer again (blue arrow), in order to monitor the dissociation rate constant.

The next question that naturally comes up is that of the limit of detection of this electronic monitoring of antigen-antibody interactions. To this end, we show in Figure 6 the kinetic recording of the current change upon switching the solution that was running through the flow cell from pure PBS buffer to a 1 nM BSA solution and then again back to pure buffer. As one can see, ΔI**_SD_** can still be monitored with a superb signal-to-noise ratio. Based on this measurement, the LOD could be estimated to be in the 100 pM analyte concentration range.

Within the Langmuir model this means that, according to Equation (1) with c**_0_** << 1 / K**_A_**, the coverage at this low concentration is given by

Θ = K_A_ c_0_ (2)

and with K_A_ = 3.3 × 10^5^ M^−1^, and c_0_ = 100 pM, this then corresponds to a coverage of bound analyte of Θ = 3.3 × 10^−5^. In other words, the minute change of the interfacial surface potential that is induced if only 1 out of 30,000 antibodies on the sensor surface binds an analyte molecule is enough to generate a current signal that can be quantified.

## 4. Food Toxin Detection, Comparison with Optical Sensing

The next example that we discuss concerns the detection of food pathogens, aflatoxins, in particular. They constitute a class of mycotoxins produced mainly by *Aspergillus flavus* and *Aspergillus parasiticus* which grow in a number of agricultural products. Aflatoxin M1 (AFM1) is the hydroxylated metabolite of aflatoxin B1 (AFB1) and can be found in urine, blood, milk, and internal organs of animals that have ingested AFB1-contaminated feed [16]. Due to its hepatotoxic and carcinogenic effects [17] and the relative stability during pasteurization or other thermal treatments, control measurements were established. For instance, the European Commission stipulates a maximum level of 50 pg·ml^−1^ for AFM1 in milk [18].

As a reference and benchmark, we start with the presentation of an optical assay based on surface-plasmon fluorescence spectroscopy, developed in our own group, combined with an inhibition immunoassay in which a derivative of AFM1 was immobilized on the sensor surface and monoclonal anti-AFM1 antibodies from the rat were used as recognition elements [19]. In this protocol the analyte sample is first incubated with a solution of anti-AFM1 antibodies of a known concentration. Some of the antibodies bind to the free AFM1 molecules; upon rinsing this cocktail through the sensor flow cell, the remaining unoccupied antibodies can then bind to the surface-immobilized AFM1 antigens, and are detected by decoration with a secondary, fluorescently labeled goat anti-rat antibody (Cy5-GaR, approximately 10.2 dyes per antibody).

**Figure 7:**
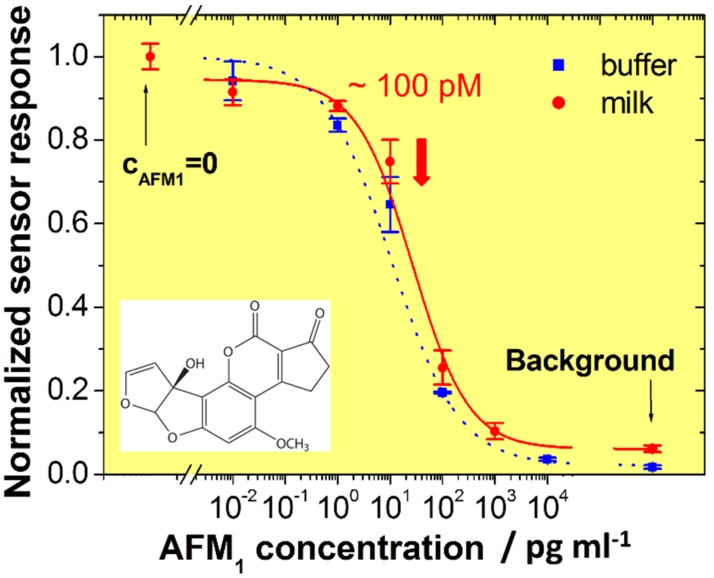
Normalized calibration curves for the detection of AFM1 in buffer (squares) and milk samples (circles).

The result of a series of measurements with sample solutions of different AFM1 concentrations is presented in Figure 7. The lower the bulk aflatoxin concentration the higher is the sensor signal from the unoccupied antibodies, now being immobilized on the sensor surface. The half-saturation value as a measure of the sensitivity of the optical assay is at c_0_ = 100 pM, with a LOD of about 0.6 pg·ml^−1^ at a processing time of 1 h.

In contrast to the optical reference assay, the electronic read-out has a number of advantages: (i) the sensor monitors the analyte binding in real time; hence is only diffusion limited and therefore faster; (ii) it gives direct kinetic information, *i.e.*, one also obtains association and dissociation rate constants; (iii) the assay does not use any secondary antibody, and hence requires fewer processing steps; (iv) the electronic sensing device based on “plastic electronics” is a disposable and does not need any sophisticated optical detection instrumentation, and hence is cheaper.

**Figure 8:**
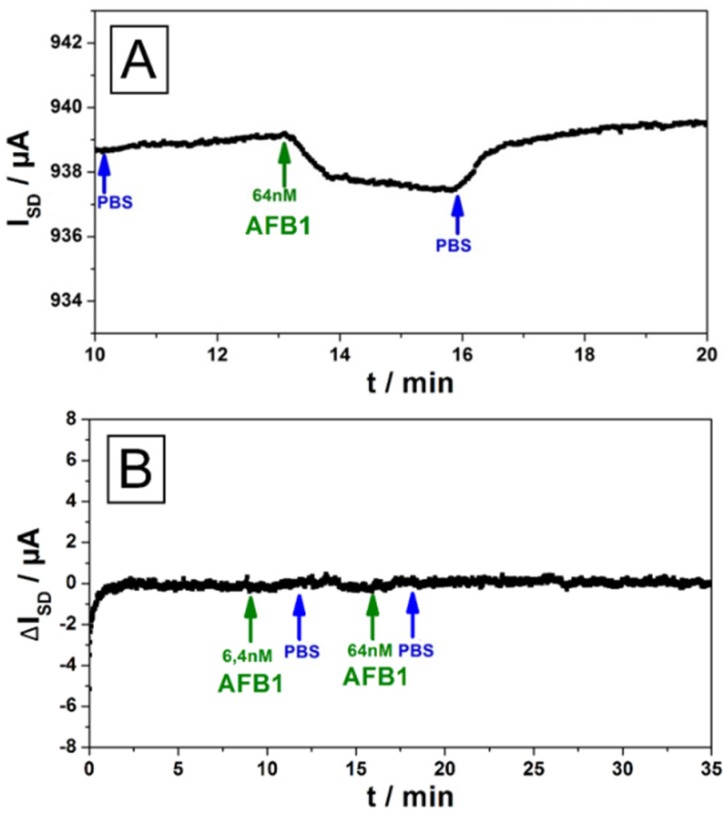
(**A**) Analysis of the binding of aflatoxin B1 (structure formula given in the inset of Figure 9) on a bare rGO gate surfaces using a blank FET. The signal strength for nanomolar concentrations was found to be only around 10% compared to the measurements were the target antibody was used (*cf.* Figure 9); (**B**) Unspecific responses for aflatoxin B1 were measured on graphene FET’s with immobilized PBSE-linker and BSA antibodies. For all tested devices no response signal was observed, indicating that no binding of AFB1 to the linker or to a non-target antibody is occurring.

We first addressed the question of non-specific binding of the analyte aflatoxin to the bare rGO gate. Figure 8A demonstrates that—Not totally unexpected given the molecular structures of the analyte molecule and graphene, respectively, suggesting a significant non-specific binding by π-π-interactions—A change in the source-drain current, I**_SD_**, upon the injection of a 64 nM solution of AFB1 into the flow cell, can indeed be monitored.

However, this non-specific binding can be totally suppressed for a test surface that is first functionalized by an antibody specific for a totally different analyte (BSA in this case). As can be seen in Figure 8B, even significant concentrations of AFB1 rinsed through the flow cell do not lead to any detectable change in the source-drain current, ΔI**_SD_**.

**Figure 9:**
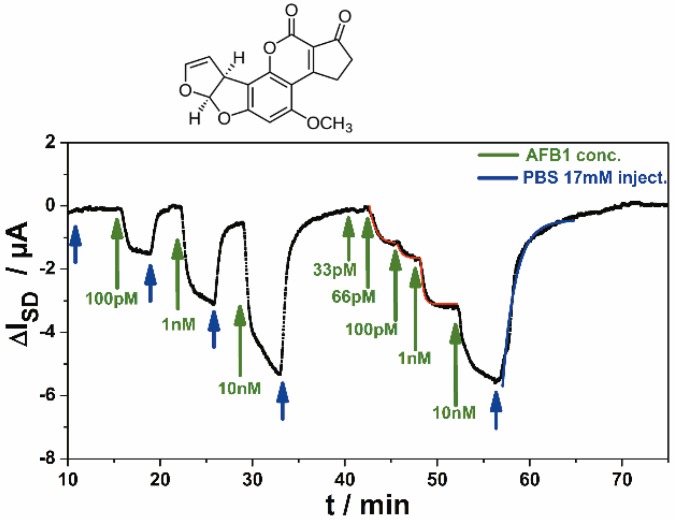
Real-time food pathogen sensor output, *i.e.*, ΔI**_SD_**, as a function of time, while AFB1 solutions of different concentrations (green arrows), alternating with pure buffer (blue arrows) were rinsed through the sample cell.

The specific binding of AFB1 to its antibody on the sensor gate surface and the sensitivity issue can be best judged by referring to the data displayed in Figure 9. Here, the antibody against AFB1, was directly immobilized on the rGO gate of the transistor. Rinsing analyte solutions of different concentrations (as indicated by the green arrows in Figure 9, alternating with pure PBS buffer, blue arrows) through the attached flow cell led to a direct sensor signal, *i.e.*, a change in the source-drain current, ΔI**_SD_**, of the FET. As one can see the sensor responds with a change of its source-drain current, with a good signal-to-noise ratio, already at analyte concentrations in the several 10 pM range. This is quite comparable to the much more demanding optical approach described in Figure 7.

A more comprehensive and quantitative evaluation of the kinetic and titration data of this electronic food toxin sensor is currently being performed by our group.

## 5. Smell Sensing

The final example that we give for the performance of the rGO FET-based biosensors concerns the development of a smell detector. Despite the enormous importance of chemical communication in nature we have essentially no technical device or sensor that could detect smells with the sensitivity and selectivity required for most applications in food quality control, for the detection of crop diseases, for medical applications like breath analysis, *etc.* Despite the fact that monitoring chemicals in chemotaxis, *i.e.*, in the search for food of many organisms or the exchange of chemicals between species as a way to communicate with each other is the oldest of our sensory repertoire, we have no sensor that offers the sensitivity and the bandwidth needed to sense and to differentiate many different odors.

The concept for a smell sensor that we are currently developing in our group is based on the functionalization of a thin film transistor with a grapheme gate by the immobilization of odorant binding proteins (OBPs) from insects as a functional element, as a bio-mimetic building block. This approach is aiming at reducing the complexity of nature to just the use of these proteins as a particularly robust element for the build-up of a first device in a bio-inspired sensor concept. OBPs from insects like those from mammals (and humans) are at the beginning of a complex amplification scheme in odorant perception that translates a first event, the binding of an odorant molecule to such an odorant binding protein, and eventually ends with the trigger of action potentials that run down the smell nerve directly into the brain.

**Figure 10:**
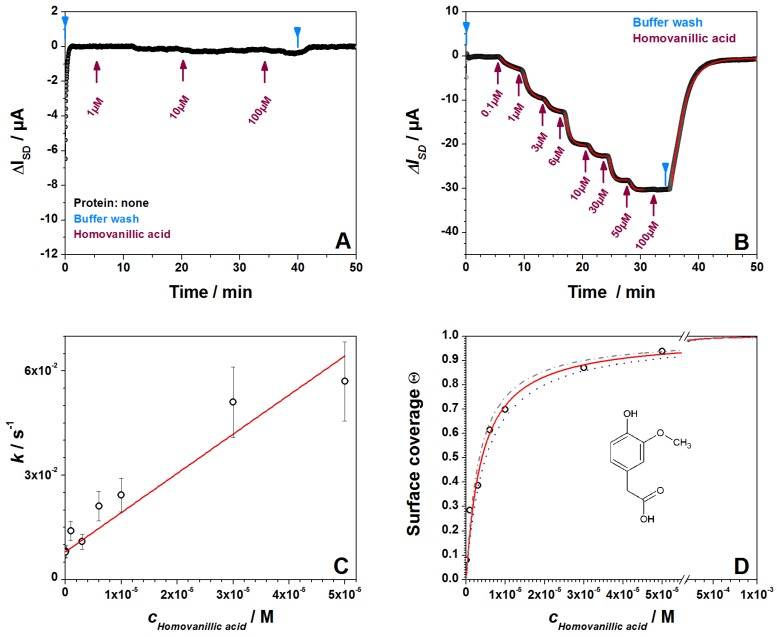
Full analysis of the recognition and binding of the odorant homovanillic acid to the odorant binding protein OBP14. (**A**) Taken with a sensor that was functionalized by the linker PBSE, however, had no protein coupled to it; (**B**) shows the global analysis for homovanillic acid binding to OPB 14 on the FET gate surface; (**C**) rate constants taken from the fits of (**B**), plotted as a function of the bilk concentration c**_0_**; (**D**) Langmuir isotherm of the titration data taken from (**B**).

Figure 10 gives a complete set of experimental data taken with a reduced graphene oxide (rGO) FET, functionalized with OBP14 from the honey bee (*A. mellifera*). The example concerns the monitoring of Homovanillic acid (structure formula given in Figure 10D), an odorant for bees which is also considered to be a tumor marker for neuroblastoma and malignant pheochromocytoma.

We start with the recordings of the change of the source-drain current from a transistor with an rGO gate that was coated with the linker molecule PBSE (*cf.* Section 2) but without an OBP coupled to it. As one can see form Figure 10A, this test for non-specific binding of the analyte leads to only a negligible signal which may result from residual π-stacking interactions between the odorant molecule and free sites on the reduced graphene oxide gate surface.

However, after the covalent immobilization of the receptor OBP14, the global analysis with both kinetic and titration information in one run results in a clear sensor signal with an excellent signal-to-noise ratio (Figure 10B). The different concentrations of the analyte solutions that were rinsed through the flow cell in this experiment are indicated by red arrows, and the flow of pure buffer, indicating the full reversibility of the ligand binding to the sensor-immobilized receptor is marked by a blue arrow.

The analysis of the kinetic data is given in Figure 10C. As predicted by the Langmuir model, the association process becomes faster with increasing bulk concentration, c**_Homovanillic_** **_acid_**. By plotting the corresponding rate constant, k, as afunction of the analyte concentration, c**_0_**, one obtains a straight line. Also, according to k = k**_on_**c**_0_** – k**_off_**, the slope of this line gives k**_on_** = 1.1 × 10^3^ M^−1^s^−1^, and the intersection with the ordinate results in k**_off_** = 8 × 10^−3^ s^−1^. This then leads to the kinetically determined affinity constant K**_A_** = k**_on_**/k**_off_** = 1.4 × 10^5^ M^−1^, corresponding to a dissociation constant K**_d_** = 7.1 µM.

This value can be compared with the data obtained from the titration experiment displayed in Figure 10D. The fit here yields K**_A_** = 2.5 × 10^5^ M^−1^ in excellent agreement with the kinetic determination. This confirms the applicability of the Langmuir model to this affinity binding reaction. It should be pointed out that by testing other ligands (odorants), dissociation constants ranging from a few µM to several mM were found, indicating the selectivity of this sensor concept based on an OBP receptor/FET bio-hybrid device [20].

## 6. Outlook

Electronic biosensing shows great promise for complementing electrochemical detection schemes on the one hand and optical concepts for the quantitative monitoring of bioanalytes on the other. In particular, the use of graphene as the conductive gate material in the preparation of thin film transistors as the sensing device offers a tremendous advantage compared to the use of organic semiconducting materials or compared to Si-based transistors, which both require far more demanding preparation protocols.

The few examples that we presented here demonstrate the versatility of the graphene-based transistor concept for biosensing in different fields of application. It should be noted that the use of monomolecular graphene oxide flakes obtained by exfoliating graphite and their reduction to reduced graphene oxide (rGO) eventually may be replaced by higher quality graphene gate materials prepared by chemical vapor deposition (which needs to be seen, though!). The basic concept, however, of functionalizing this gate material by different types of biorecognition elements, receptors, antibodies, *etc.*, able to bind the analyte molecule of interest, has proven already to yield the sensitivity and selectivity needed for practical applications of these devices.

Some of the fundamental issues associated with rGO FET-based biosensing still need further work and clarification; e.g., the role of local pH changes, direct but also indirect modifications of the surface potential at the gate-electrolyte interface by variations of the local ionic milieu, the concentration, profile and chemical nature of ions and counter-ions associated with the biorecognition reaction, the role of dipole potentials, *etc.*, are far from being completely understood. Other than in optical biosensing, this also gives the concept of anti-fouling coatings a different meaning: it is absolutely not clear how to best minimize non-specific binding. What determines eventually the limit of detection in rGO-based electronic biosensing? What is the relationship between the gate architecture and the minimal surface coverage that is needed in order to sense an electronic signal? Would eventually a single occupied site on the biofunctionalized gate surface be enough to modify the source-drain current?

A final comment refers to the last application, *i.e.*, the sensing of smells. The example given was based on monitoring of changes induced in an odorant binding protein immobilized on the gate surface of the transistor by the binding of a ligand, an odorant, from the aqueous phase of the respective analyte solution in contact with the sensor. This format mimics to some extend the situation in the nose of vertebrates where the sensory neurons and the associated odorant binding proteins are covered and, hence, protected and hydrated by the mucosa. It also reflects properly the situation in the sensilla of the insect antennae, where the OBPs are in the lymph, *i.e.*, also in an aqueous environment. In that sense, the presented results are quite relevant also for the development of a smell sensor. In fact, a direct comparison between the sensitivity of a whole insect antenna from an insect, the red flour beetle Tribolium castaneum, responding to a certain partial pressure of an odorant in a carrier gas, with that of a rGO transistor functionalized by one of the key OBPs from this species, responding to an aqueous solution of the same odorant, gave very comparable results [21]. Of course, our final goal is to operate these devices also in air, which seems to be absolutely possible by using hydrogels as a protective coating of the device, mimicking the mucosa that keeps all the biocomponents of the sensor in a hydrated and therefore functional environment.
